# Phthalate Metabolites in Urine of Chinese Children and Their Association with Asthma and Allergic Symptoms

**DOI:** 10.3390/ijerph192114083

**Published:** 2022-10-28

**Authors:** Yuxuan Zhao, Yuexia Sun, Changqi Zhu, Ying Zhang, Jing Hou, Qinghao Zhang, Yeganeh Ataei

**Affiliations:** 1 Tianjin Key Laboratory of Indoor Air Environmental Quality Control, School of Environmental Science and Engineering, Tianjin University, Tianjin 300350, China; 2 School of Environmental Science and Engineering, Tianjin University, Tianjin 300350, China

**Keywords:** allergy, biomarkers, endocrine disruptors, phthalate metabolites

## Abstract

Phthalates are ubiquitous ‘modern’ chemical compounds with potential negative impacts on children’s health. A nested case–control study was designed to investigate associations of phthalate exposure with children’s asthma and allergic symptoms. We collected 243 first morning urine samples from 4–8-year-old children in Tianjin, China. Eight metabolites (i.e., mono-ethyl phthalate (MEP), mono-isobutyl phthalate (MiBP), mono-n-butyl phthalate (MnBP), mono-benzyl phthalate (MBzP) and mono-2-ethylhexyl phthalate (MEHP), mono-(2-ethyl-5-carboxylpentyl) phthalate (MECPP), mono-(2-ethyl-5-hydroxyhexyl) phthalate (MEHHP) and mono-(2-ethyl-5-oxohexyl) phthalate (MEOHP)) of five phthalates were analyzed using HPLC-MS. MiBP, MnBP and MECPP were the dominant phthalate metabolites in urine of children in Tianjin with median concentrations of 31.6 μg/L, 26.24 μg/L and 46.12 μg/L, respectively. We found significantly positive associations of diagnosed asthma with MnBP (adjusted odds ratios (AOR): 1.96; 95% confidence intervals (CIs): 1.07–3.61), MEHHP (AOR: 2.00; 95% CI: 1.08–3.71) and MEOHP (AOR: 2.09; 95% CI: 1.06–4.10). Our study indicates that phthalate exposure in childhood, especially to di-n-butyl phthalate (DnBP) and di(2-ethylhexyl) phthalate (DEHP), may be a risk factor for children’s asthma.

## 1. Introduction

The prevalence of asthma and allergies has increased throughout the world during the last few decades [1], imposing a burden on patients’ quality of life. Although the underlying causal factors for the increase remain unknown, studies have shown that indoor pollutants are an important factor [2]. Phthalates, which are ‘modern’ chemical compounds, have been detected ubiquitously in indoor environment [3]. Several epidemiological studies [2,4,5] have suggested that exposure to phthalates may be a risk factor for asthma and allergy.

Phthalates are widely used as plasticizers and stabilizers in daily consumer products [6,7] such as building materials, furniture, food packaging, children’s toys, cosmetics and air fresheners. Since phthalates are not covalently bonded to the product, they are easily released into the environment and can be found in air [6], dust [8], food [9] and water [10]. People are mainly exposed to phthalates via ingestion, inhalation and dermal absorption [11]. Phthalates are considered endocrine disruptors and may cause damage to the reproductive [12], respiratory [13], nervous [14] and metabolic systems [15]. Studies with animal and in vitro models have shown that phthalates have immunomodulatory properties [16,17].

Researchers have collected dust to investigate the association of phthalates with asthma and allergic symptoms [2,4,5]. However, phthalate metabolites in urine are more suitable to reflect combined exposure due to the variety of exposure routes. Although many studies have examined the association between phthalate metabolites and adverse health effects, there is limited research on the association of phthalate metabolites with asthma and allergic symptoms. Additionally, these studies have not been consistent, with some finding a positive association between phthalate metabolites and allergies [18,19] and others finding no significant association [20].

The aim of the present study is to evaluate the phthalates exposure levels through urinary metabolites in Chinese children, and to investigate the potential associations of asthma and allergic symptoms with the concentrations of different phthalate metabolites in urine.

## 2. Materials and Methods

### 2.1. Study Design

This study was part of the CCHH (China, Children, Homes, Health) study in the Tianjin region. The CCHH study was based on the questionnaire and protocols developed for ‘Dampness in Buildings and Health’ (DBH) study in Sweden [21], and subsequently used in global investigations [22]. In phase I (May 2013–December 2014), questionnaires were sent to primary schools and daycare centers, where parents responded to questionnaires to provide information about their children aged 0–8 years. The questionnaire included demographic information about the child and family, the health status of the child and family members, living conditions and food habits of the child. The second phase (September 2013–January 2016), a case–control study [23], involved environmental inspections and measurements of 399 homes, as well as the collection of bedroom dust and urine samples from children. This paper is based on the research of the second phase.

### 2.2. Health Outcomes

Asthma and allergic symptoms among children were self-reported by parents through the questionnaire survey. These questions were identical to those used in the ISAAC study [24]. Children with asthma and/or allergy were classified as cases, while those without any allergic symptoms were classified as controls. The definitions of asthma and allergic symptoms were as follows.

- Asthma (Wheezing ever: “has your child ever had wheezing or whistling in the chest at any time in the past?” Wheezing current: “in the last 12 months has your child had wheezing or whistling in the chest?” Dry cough current: “in the last 12 months has your child had a dry cough at night for more than two weeks, apart from a cough associated with a cold or chest infection?” Diagnosed asthma: “has your child been diagnosed with asthma by a doctor?”).
- Rhinitis (Rhinitis ever: “has your child ever had a problem with sneezing, or a runny, or a blocked nose when he/she did not have a cold or a flu?” Rhinitis current: “in the last 12 months has your child had a problem with sneezing, or a runny, or a blocked nose when he/she did not have a cold or the flu?” Diagnosed rhinitis: “has your child been diagnosed with hay fever or allergic rhinitis by a doctor?”).
- Eczema (Eczema ever: “has your child ever had an itchy rash coming and going for at least 6 months?” Eczema current: “in the last 12 months has your child had eczema symptoms at any time?” Diagnosed eczema: “has your child been diagnosed with eczema by a doctor?”) [23].

This study was approved by the research office at Tianjin University. Guardians of participants provided written informed consent to participate in this study.

### 2.3. Urine Collection and Phthalate Metabolites Analysis

A 50 mL beaker and two cryotubes were given to the parents for the collection of their child’s urine. The first morning urine sample was collected by parents and stored in the refrigerator until the home inspection. Contact with plastics during the process was avoided. The urine samples were transported back to the laboratory by the home inspectors and stored in a refrigerator at −20 °C until analysis.

The target phthalate monoesters were mono-ethyl phthalate (MEP), mono-isobutyl phthalate (MiBP), mono-n-butyl phthalate (MnBP), mono-benzyl phthalate (MBzP) and mono-2-ethylhexyl phthalate (MEHP), mono-(2-ethyl-5-carboxylpentyl) phthalate (MECPP), mono-(2-ethyl-5-hydroxyhexyl) phthalate (MEHHP) and mono-(2-ethyl-5-oxohexyl) phthalate (MEOHP). MEP, MiBP, MnBP, MBzP and MEHP were purchased from AccuStandard Inc. (New Haven, CT, USA), with a purity of 95%. MECPP, MEHHP and MEOHP (95%) were purchased from TRC Inc. (Montreal, QC, Canada). 13C-labeled internal standards (^13^C_4_-MnBP, ^13^C_4_-MEP and ^13^C_4_-MECPP) with a purity of 99% were purchased from Cambridge Isotope Laboratories Inc. (Tewksbury, MA, USA). Methanol and acetonitrile (HPLC grade) were purchased from Fisher Inc. (Waltham, MA, USA). Formic acid, glacial acetic acid, ammonium acetate and sodium bicarbonate (HPLC-grade) were purchased from Chemicals Northwest Inc. (Berlin, Germany). β-glucuronidase (95%) was purchased from Sigma Inc. (Roedermark, Germany).

The urine samples were analyzed at Nankai University, China. Each urine sample was thawed, and an aliquot (0.5 mL) of urine was spiked with ammonium acetate (200 μL, pH 4.5), β-glucuronidase (75 μL, 250 unit/mL), a mixture of isotope internal standards (100 μL, 250 μg/L), and 0.5 mL Milli-Q water, followed by incubation at 37 °C overnight. After enzymatic hydrolysis, the urine samples were concentrated and cleaned using CNW Poly-Sery MAX SPE cartridges (60 mg/3 mL, CNW Technologies GmbH, Düsseldorf, Germany). The cartridges were conditioned with 3 mL of methanol and 3 mL of Milli-Q water. After the urine samples were loaded onto the cartridges, the cartridges were rinsed with 3 mL of Milli-Q water, 3 mL of sodium bicarbonate buffer and 3 mL methanol. The analytes were eluted with 5 mL of methanol containing 2% formic acid (*v*/*v*). Then, the eluate was concentrated to near dryness under a gentle stream of nitrogen in a 45 °C water bath and reconstituted in 0.5 mL of 10% acetonitrile aqueous solution (*v*/*v*). An HPLC-MS (high-performance liquid chromatography-mass spectrometry) method was established to analyze concentrations of phthalate metabolites in the urine samples. A KinetexR 1.7 μm Phenyl-Hexyl column was used at 40 °C for chromatographic fractionation. A linear gradient program was used with an organic solvent (acetonitrile and 0.1% acetic acid) and an aqueous solution containing 0.1% acetic acid at a flow rate of 0.4 mL/min. Agilent G6460 was used for the mass spectrometry, and ESI was used as the electrospray ion source in negative ion mode.

Mass-charge ratio and retention time (shown in Table S1) were used for qualification, and an internal standard method was used for quantitation. The limits of detection (LOD) were the concentrations at a signal-noise ratio of 3:1. The LOD for MEP, MiBP, MnBP, MBzP, MEHP, MECPP MEHHP and MEOHP were 1.224 μg/L, 0.109 μg/L, 0.256 μg/L, 0.176 μg/L, 0.0733 μg/L, 0.091 μg/L, 0.090 μg/L and 0.128 μg/L, respectively. The average recoveries for eight phthalate metabolites ranged from 80% to 120%, and the relative standard deviation of the spiked samples ranged from 2.0% to 7.5%. If the concentration of phthalate metabolites was lower than the LOD, then half values of the LOD were nominated as the exposure level and used for further statistical analysis.

### 2.4. Statistical Analysis

In terms of the atopic march, children under the age of 4 may not yet show symptoms of asthma or rhinitis. The inclusion of children under 4 years in this study could obscure an association between phthalate metabolites and allergy. Therefore, the statistical analysis focused on children aged 4–8 years. The concentrations of phthalate metabolites in urine were not log-normally distributed. The concentrations were reported as minimum, maximum, means, medians, 25% quartiles and 75% quartiles. Mann–Whitney U-tests were conducted to analyze the different distributions of phthalate metabolites concentration between children with and without asthma and allergic symptoms. Then, logistic regression models were performed with phthalate metabolites as continuous variables. The influence of phthalate exposure on asthma and allergic symptoms was presented as odds ratios (OR) with 95% confidence intervals (CIs) for an interquartile range increase in concentrations of phthalate metabolites. Odds ratios were adjusted for the child’s age, gender, family allergic history, home dampness indicators, environmental tobacco smoke exposure and measurement seasons. Sensitivity analysis was performed by stratifying for gender and measurement season. *p*-values less than 0.05 were accepted as significant. Statistical analysis was performed with SPSS 26.

## 3. Results

### 3.1. Demographic Information of Investigated Children and Their Health Outcomes

A total of 243 children aged 4–8 years with complete health information were used for subsequent analysis. Among these 243 children, 49% were boys, 72.4% were preschool children (≤6 years old), and 33.8% had a family allergic history. Thirteen (13) children had both asthma and rhinitis symptoms, ten (10) had both asthma and eczema symptoms, fifty-seven (57) had both rhinitis and eczema symptoms and fifty-three (53) had these three symptoms. Demographic information, home environmental factors and measurement seasons of the investigated children for different health outcomes are shown in Table 1.

### 3.2. Concentrations of Phthalate Metabolites in Urine

The distribution of phthalate metabolites in urine is shown in Table 2. The detection rates of phthalate metabolites in urine were all above 90%, except for MBzP. MBzP was detected in only 8.4% of urine samples. Due to the low detection rate of MBzP, it was not considered in the subsequent analysis. MiBP, MnBP and MECPP were the main phthalate metabolites of children in the Tianjin area. We found significant differences in the seasonal distributions of phthalate metabolite concentrations (Supplementary Table S2).

### 3.3. Associations between Phthalate Metabolites in Urine and Health Outcomes

Median concentrations of phthalate metabolites for children with and without allergic symptoms are presented in Table 3. In general, metabolites of DnBP and DEHP had higher concentrations among allergic children, especially among children with asthma and rhinitis symptoms. Urine concentrations of MiBP and MEOHP among children who had wheezing in the last 12 months were significantly higher than those without wheezing. Children with diagnosed asthma had significantly higher concentrations of MnBP, MEHHP and MEOHP compared to those without diagnosed asthma.

Crude and adjusted associations of phthalate metabolites with children’s allergic symptoms are shown in Supplementary Table S3 and Table 4, respectively. We found significantly positive associations of diagnosed asthma with MnBP (AOR: 1.96; 95% CI: 1.07–3.61), MEHHP (AOR: 2.00; 95% CI: 1.08–3.71) and MEOHP (AOR: 2.09; 95% CI: 1.06–4.10). The sensitivity analysis stratifying for gender is shown in Table 5 and Table 6. We found significantly positive associations of diagnosed asthma with MnBP, MEHP, MEHHP and MEOHP in boys. In girls, positive but non-significant associations of diagnosed asthma with MnBP, MEHHP and MEOHP were also found. Associations between MEHP and eczema symptoms, MECPP and rhinitis symptoms showed different patterns in boys and girls. MEHP and MECPP seemed to be at greater risk for eczema and rhinitis symptoms among girls. Stratification analysis between winter–spring and summer–autumn are shown in Supplementary Table S4. The higher exposure to metabolites of DnBP and DEHP was still a risk factor for diagnosed asthma in both winter–spring and summer–autumn, even though the association became less significant (*p* > 0.05). It was interesting to find a significant positive association between MECPP and eczema current (AOR: 1.04–5.98) in the summer–autumn season.

## 4. Discussion

The main phthalate metabolites detected in this study were MiBP, MnBP and MECPP, which is consistent with findings of other studies in China [25,26,27]. Similar to the results of this study, the detection rates of MBzP in previous Chinese studies were also very low. The concentrations of MBzP (metabolites of benzyl butyl phthalate (BBzP)) detected in this study were lower than those in other countries (see Supplementary Table S5). This is consistent with the low levels of BBzP in these children’s bedroom dust [3]. It is possible that China’s policy (GB9685-2008) restricting additives for food containers and packing materials in 2008 has led to the reduction in urinary MBzP [3]. The concentrations of MiBP, MnBP and MEOHP in this study were lower than those found in Shanghai and Beijing studies (Supplementary Table S5). This may reflect regional differences among Tianjin, Beijing and Shanghai.

We found significant associations of diagnosed asthma with MEHHP and MEOHP, the metabolites of di(2-ethylhexyl) phthalate (DEHP). Associations of other asthma-related symptoms with MEHHP and MEOHP were also seen, although these associations did not reach statistical significance. DEHP was found to be a risk factor for asthma and allergic symptoms in a case–control study in Sweden, and the relationship between diagnosed asthma and DEHP was a dose–response relationship [4]. The sum of DEHP metabolites in urine has also been found to be associated with diagnosed asthma among 14–15-year-olds in Belgium [28]. In addition, a longitudinal study in Spain concluded that prenatal exposure to DEHP might increase the risk of asthma symptoms and respiratory tract infections throughout childhood [29]. The potential mechanism is the adjuvant effect of phthalates on the immune response [30,31]. It is suspected that phthalates promote Th2 differentiation and influence the antibody response [16,17]. DEHP has also been described to alter airway cell differentiation and lung surfactant protein production [32].

We found the first (MEHP) and the second (MEHHP and MEOHP) metabolites of DEHP were positively associated with diagnosed asthma and related symptoms. However, no consistent patterns were seen between asthma symptoms and MECPP, another DEHP metabolite. In an American study, the total metabolites of DEHP were found to be significantly and negatively associated with current asthma among children aged 6–17 years [33]. Moreover, in our study, MECPP was a greater risk factor for rhinitis in girls. In view of the harmful health effects of DEHP on humans, future studies on the toxicity of its metabolites are necessary.

We found that MnBP was a risk factor for asthma symptoms, especially for diagnosed asthma, which reached significance. This is consistent with previous studies. A Chinese study in Shanghai similarly found that MnBP in the highest quartile was associated with an increased risk of wheezing, rhinitis and eczema in children [19]. Moreover, prenatal metabolites of DnBP have been found to be associated with the diagnosis of current asthma in children at 5–11 years [34].

In gender-stratified analyses, higher concentrations of MnBP, MEHHP, and MEOHP were related to diagnosed asthma among both boys and girls. These health effects of metabolites reached a significant level for boys. Previous limited data on the associations of phthalate exposure with asthma and allergy among different genders did not reach consistent conclusions. Some studies reported no gender modification [19], others found a higher risk of asthma in girls associated with phthalate exposure [18,29,35], while still others found a higher risk in boys [36]. Gender-specific effects of phthalate exposure on asthma need to be further investigated.

### Strengths and Limitations

A strength of our study is the large sample size. The questions about health outcomes in the ISAAC questionnaire have been validated [37]. Moreover, we have collected detailed environmental factors in homes of the investigated children, which makes it possible to control for multiple potential confounders. Therefore, our study should have strong internal validity.

We observed consistent positive associations of MnBP, MEHHP and MEOHP with asthma and related symptoms in multiple tests. Moreover, the positive association between these metabolites and diagnosed asthma reached a significant level. We believe this finding is more than just chance association.

Our measurements on phthalate metabolites were based on a single urine sample. Hauser et al. [38] have suggested that a single urine sample was moderately predictive. Teitelbaum et al. [39] observed relatively stable concentrations of phthalate metabolites among spot urine samples from children over a period of 6 months. Therefore, it was reasonable for this study to use a single morning urine sample. However, we still have a limitation of not adjusting for urine dilution. Nevertheless, all samples were first morning urine, which may have reduced the differences in urine specific gravity between samples. Despite this limitation, we found strong associations between diagnosed asthma and MnBP, MEHHP and MEOHP.

## 5. Conclusions

MiBP, MnBP and MECPP are the main phthalate metabolites of children in Tianjin area. Our data indicate that phthalate exposure in childhood, especially to DEHP and DnBP, may be a risk factor for children’s asthma. Further studies are needed to confirm our findings.

## Figures and Tables

**Table 1:** Demographic information, home environmental factors and measurement seasons for different health outcomes of the investigated children 4–8 years old in Tianjin, China.

		Wheeze Ever		Wheeze Current		Current Dry Cough		Diagnosed Asthma		Rhinitis Ever		Rhinitis Current		Diagnosed Rhinitis		Eczema Ever		Eczema Current		Diagnosed Eczema	
	(%)	No, 194	Yes, 49	No, 218	Yes, 25	No, 196	Yes, 47	No, 206	Yes, 37	No, 97	Yes, 146	No, 119	Yes, 124	No, 187	Yes, 56	No, 109	Yes, 134	No, 176	Yes, 67	No, 114	Yes, 129
Gender	Male	47.6	54.2	47.2	64.0	47.9	53.2	47.8	55.6	46.8	50.3	50.0	48.0	49.5	47.3	50.5	47.7	48.3	50.7	48.7	49.2
	Female	52.4	45.8	52.8	36.0	52.1	46.8	52.2	44.4	53.2	49.7	50.0	52.0	50.5	52.7	49.5	52.3	51.7	49.3	51.3	50.8
Age	4–6	72.2	73.5	72.0	76.0	68.4	89.4	71.8	75.7	61.9	79.5	65.5	79.0	70.1	80.4	69.7	74.6	72.2	73.1	69.3	75.2
	7–8	27.8	26.5	28.0	24.0	31.6	10.6	28.2	24.3	38.1	20.5	34.5	21.0	29.9	19.6	30.3	25.4	27.8	26.9	30.7	24.8
Allergic history	Yes	30.4	47.8	31.0	58.3	32.8	37.8	30.0	55.9	30.2	36.2	28.8	38.7	25.1	63.0	28.7	38.0	31.0	40.9	30.1	37.1
Dampness	Yes	24.6	32.7	25.1	36.0	25.3	30.4	24.1	37.8	24.2	27.7	23.1	29.4	24.9	30.9	19.0	32.1	24.6	30.8	22.0	29.9
Environmental tobacco exposure	Yes	44.3	52.1	45.4	50.0	45.1	48.9	44.7	52.8	47.4	44.8	46.2	45.5	45.5	47.3	45.9	45.9	47.7	40.9	46.5	45.3
Measurement season	Spring	14.5	22.4	15.2	24.0	14.3	23.9	15.6	18.9	7.2	22.1	9.2	22.8	15.5	18.2	14.8	17.2	15.4	17.9	12.4	19.4
	Summer	16.1	16.3	16.6	12.0	18.4	6.5	16.1	16.2	18.6	14.5	16.8	15.4	16.0	16.4	18.5	14.2	17.1	13.4	20.4	12.4
	Autumn	27.5	24.5	26.7	28.0	27.6	23.9	28.3	18.9	32.0	23.4	27.7	26.0	28.3	21.8	23.1	29.9	26.9	26.9	24.8	28.7
	Winter	42.0	36.7	41.5	36.0	39.8	45.7	40.0	45.9	42.3	40.0	46.2	35.8	40.1	43.6	43.5	38.8	40.6	41.8	42.5	39.5

**Table 2:** Distribution of phthalate metabolite concentrations (μg/L) in urine samples for 294 children aged 4–8 years old in Tianjin, China.

	MEP	MiBP	MnBP	MBzP	MEHP	MECPP	MEHHP	MEOHP
Min	ND	ND	0.33	ND	ND	ND	ND	ND
Mean	38.51	27.88	29.45	0.11	7.58	37.57	13.71	9.06
Max	1095.78	96.85	147.98	1.05	15.77	164.97	68.82	30.48
25%	5.78	22.64	3.14	0.09	5.33	12.51	4.52	4.21
50%	18.36	31.60	26.24	0.09	7.50	46.12	16.71	9.61
75%	50.20	33.54	44.94	0.09	9.36	55.86	18.73	13.62
Detection rate	96.20%	99.50%	100%	8.40%	99.50%	96.80%	95.70%	92.00%

ND—the concentration of phthalate metabolites was lower than the LOD.

**Table 3:** Median concentrations (μg/L) of phthalate metabolites among children with and without allergic symptoms.

		MEP	MiBP	MnBP	MEHP	MECPP	MEHHP	MEOHP
Wheeze ever	No	19.41	31.69	24.74	7.42	46.09	16.71	9.60
	Yes	16.32	31.58	34.49	8.12	47.81	16.61	13.10
*p* ^a^		0.94	0.37	0.26	0.28	0.87	0.53	0.13
Wheeze current	No	18.35	31.51	24.77	7.49	47.34	16.51	9.57
	Yes	21.99	32.74	37.81	8.23	27.52	18.52	13.76
*p* ^a^		0.37	**0.04**	0.20	0.31	0.13	0.08	**0.01**
Current dry cough	No	17.02	31.72	26.07	7.63	42.86	16.67	9.61
	Yes	21.97	31.46	26.34	6.27	53.15	16.84	10.65
*p* ^a^		0.16	0.69	0.65	0.63	0.61	0.64	0.55
Diagnosed asthma	No	19.41	31.59	24.30	6.39	47.25	16.63	9.57
	Yes	15.75	32.65	39.85	8.35	43.38	17.39	13.55
*p* ^a^		0.97	0.09	**0.03**	0.09	0.92	**0.05**	**0.01**
Rhinitis ever	No	19.41	31.74	25.99	7.42	41.82	16.71	9.61
	Yes	18.35	31.58	26.25	7.67	47.30	16.65	10.66
*p* ^a^		0.94	0.76	0.91	0.91	0.96	0.57	0.67
Rhinitis current	No	19.43	31.65	27.69	6.91	39.66	16.80	10.15
	Yes	18.29	31.56	26.11	7.75	49.46	16.28	8.49
*p* ^a^		0.62	0.78	0.63	0.53	0.42	0.23	0.61
Diagnosed rhinitis	No	18.36	31.51	26.02	7.49	45.03	16.72	9.64
	Yes	20.16	32.11	30.10	8.07	47.34	16.50	8.49
*p* ^a^		0.57	0.22	0.66	0.55	0.89	0.84	0.65
Eczema ever	No	21.25	31.92	29.98	7.69	37.53	17.04	10.68
	Yes	17.00	31.46	25.99	7.43	50.73	16.19	8.49
*p* ^a^		0.11	0.08	0.51	0.44	0.20	**0.04**	0.18
Eczema current	No	19.45	31.70	25.99	7.65	43.90	16.84	10.66
	Yes	16.40	31.51	27.60	6.36	49.04	16.25	8.47
*p* ^a^		0.19	0.31	0.89	0.41	0.51	0.22	0.09
Diagnosed eczema	No	19.41	31.70	26.31	7.55	41.74	16.90	10.66
	Yes	17.65	31.58	26.18	7.46	47.85	16.32	9.02
*p* ^a^		0.31	0.73	0.67	0.96	0.82	0.31	0.35

^a^ *p*-values were calculated by Mann–Whitney U-test. *p*-value less than 0.05 are indicated in bold for significance.

**Table 4:** Adjusted odds ratios (AOR) with 95% CIs of phthalate metabolites with children’s asthma and allergy in multivariate regression models.

	MEP	MiBP	MnBP	MEHP	MECPP	MEHHP	MEOHP
Wheeze ever	1.13 (0.94–1.36)	0.98 (0.75–1.28)	1.38 (0.80–2.38)	1.14 (0.70–1.84)	0.95 (0.51–1.79)	1.09 (0.66–1.79)	1.31 (0.75–2.30)
Wheeze current	1.17 (0.96–1.42)	1.16 (0.82–1.62)	1.46 (0.71–2.98)	1.45 (0.76–2.77)	0.55 (0.23–1.32)	1.17 (0.63–2.17)	1.79 (0.83–3.86)
Current dry cough	0.99 (0.82–1.19)	1.09 (0.84–1.43)	1.17 (0.65–2.10)	0.98 (0.61–1.60)	0.95 (0.50–1.78)	1.41 (0.84–2.37)	1.28 (0.71–2.29)
Diagnosed asthma	0.97 (0.74–1.26)	1.16 (0.86–1.56)	**1.96 (1.07–3.61)**	1.54 (0.88–2.67)	0.89 (0.43–1.83)	**2.00 (1.08–3.71)**	**2.09 (1.06–4.10)**
Rhinitis ever	1.03 (0.87–1.21)	0.93 (0.75–1.16)	1.06 (0.67–1.66)	1.01 (0.69–1.49)	0.77 (0.46–1.29)	0.85 (0.56–1.29)	1.02 (0.65–1.61)
Rhinitis current	0.90 (0.75–1.09)	0.94 (0.76–1.17)	0.96 (0.61–1.51)	1.08 (0.74–1.59)	0.98 (0.59–1.62)	0.71 (0.46–1.09)	0.76 (0.48–1.19)
Diagnosed rhinitis	0.75 (0.44–1.26)	1.03 (0.78–1.36)	1.18 (0.67–2.09)	1.09 (0.66–1.80)	0.95 (0.51–1.80)	1.10 (0.66–1.82)	0.91 (0.52–1.61)
Eczema ever	0.85 (0.67–1.08)	0.88 (0.71–1.10)	0.82 (0.52–1.28)	0.81 (0.55–1.19)	1.23 (0.74–2.05)	0.79 (0.52–1.20)	0.78 (0.49–1.22)
Eczema current	0.89 (0.67–1.18)	0.90 (0.71–1.15)	0.92 (0.56–1.53)	0.75 (0.48–1.16)	1.39 (0.80–2.43)	0.71 (0.44–1.14)	0.64 (0.39–1.06)
Diagnosed eczema	0.90 (0.74–1.09)	1.04 (0.84–1.29)	0.98 (0.63–1.52)	1.08 (0.74–1.57)	0.92 (0.56–1.52)	0.93 (0.62–1.40)	0.85 (0.55–1.34)

Odds ratios were adjusted for age, gender, allergic history, home dampness, environmental tobacco smoke exposure and measurement season. Concentrations of phthalate metabolites were coded as continuous variables with an interquartile increase step. *p*-value less than 0.05 are indicated in bold for significance.

**Table 5:** Associations [AOR (95% CI)] between urinary phthalate metabolite concentrations and allergic symptoms in boys (*n* = 117).

	MEP	MiBP	MnBP	MEHP	MECPP	MEHHP	MEOHP
Wheeze ever	1.15 (0.90–1.46)	1.00 (0.68–1.47)	1.46 (0.65–3.30)	1.03 (0.48–2.21)	0.78 (0.30–2.05)	1.03 (0.50–2.10)	1.44 (0.58–3.57)
Wheeze current	1.26 (0.87–1.82)	1.27 (0.84–1.92)	1.49 (0.58–3.81)	1.88 (0.72–4.95)	0.64 (0.21–2.01)	1.38 (0.61–3.10)	2.77 (0.81–9.48)
Current dry cough	0.99 (0.78–1.24)	1.40 (0.93–2.11)	1.78 (0.75–4.20)	0.73 (0.36–1.49)	1.35 (0.50–3.66)	1.74 (0.79–3.84)	1.25 (0.51–3.08)
Diagnosed asthma	0.97 (0.72–1.31)	1.34 (0.89–2.01)	**2.76 (1.08–7.06)**	**2.70 (1.10–6.61)**	0.54 (0.19–1.60)	**3.50 (1.11–11.02)**	**4.63 (1.30–16.50)**
Rhinitis ever	1.43 (0.72–2.84)	0.81 (0.58–1.13)	1.10 (0.54–2.22)	0.91 (0.51–1.63)	**0.39 (0.17–0.93)**	0.93 (0.51–1.70)	1.05 (0.51–2.19)
Rhinitis current	0.92 (0.75–1.13)	0.85 (0.61–1.18)	0.99 (0.49–2.02)	0.97 (0.53–1.76)	**0.41 (0.17–0.97)**	0.86 (0.47–1.59)	0.76 (0.36–1.61)
Diagnosed rhinitis	0.98 (0.70–1.37)	1.04 (0.69–1.56)	1.66 (0.74–3.72)	0.95 (0.45–2.02)	0.53 (0.19–1.43)	1.07 (0.53–2.16)	0.92 (0.38–2.24)
Eczema ever	0.95 (0.77–1.17)	0.86 (0.62–1.20)	0.69 (0.35–1.35)	0.56 (0.31–1.03)	0.85 (0.39–1.88)	0.66 (0.36–1.20)	0.58 (0.28–1.21)
Eczema current	0.94 (0.70–1.25)	0.80 (0.55–1.17)	0.75 (0.35–1.60)	**0.47 (0.23–0.96)**	1.28 (0.54–3.06)	0.58 (0.28–1.17)	0.48 (0.21–1.06)
Diagnosed eczema	0.93 (0.751.15)	0.92 (0.66–1.27)	0.74 (0.37–1.49)	0.71 (0.39–1.30)	0.77 (0.34–1.74)	0.72 (0.39–1.31)	0.56 (0.26–1.19)

All models were adjusted for age, allergic history, home dampness, environmental tobacco smoke exposure and measurement season. *p*-value less than 0.05 are indicated in bold for significance.

**Table 6:** Associations [AOR (95% CI)] between urinary phthalate metabolite concentrations and allergic symptoms in girls (*n* = 122).

	MEP	MiBP	MnBP	MEHP	MECPP	MEHHP	MEOHP
Wheeze ever	1.01 (0.66–1.55)	0.89 (0.58–1.37)	1.13 (0.52–2.47)	1.20 (0.61–2.38)	1.26 (0.57–2.78)	1.16 (0.51–2.65)	1.15 (0.51–2.60)
Wheeze current	0.30 (0.05–1.91)	0.99 (0.49–2.00)	1.58 (0.44–5.69)	1.40 (0.48–4.09)	0.52 (0.14–1.94)	1.19 (0.35–4.07)	1.25 (0.37–4.29)
Current dry cough	1.09 (0.73–1.62)	0.81 (0.53–1.25)	0.79 (0.33–1.89)	1.38 (0.68–2.79)	0.63 (0.26–1.55)	1.11 (0.47–2.61)	1.31 (0.56–3.09)
Diagnosed asthma	0.79 (0.37–1.68)	0.84 (0.49–1.42)	1.14 (0.44–3.00)	0.79 (0.34–1.82)	1.52 (0.59–3.89)	1.24 (0.46–3.32)	1.09 (0.41–2.90)
Rhinitis ever	0.78 (0.54–1.12)	1.09 (0.78–1.52)	1.01 (0.56–1.84)	1.14 (0.65–1.97)	1.22 (0.62–2.39)	0.78 (0.41–1.51)	0.98 (0.52–1.86)
Rhinitis current	0.79 (0.54–1.14)	1.03 (0.74–1.42)	0.87 (0.47–1.58)	1.17 (0.68–2.01)	1.89 (0.94–3.80)	0.53 (0.27–1.06)	0.70 (0.37–1.33)
Diagnosed rhinitis	0.42 (0.15–1.20)	1.08 (0.69–1.68)	0.81 (0.33–1.96)	1.29 (0.63–2.62)	1.41 (0.64–3.10)	1.31 (0.57–2.99)	0.95 (0.41–2.17)
Eczema ever	0.67 (0.43–1.04)	0.88 (0.62–1.23)	0.90 (0.49–1.64)	1.08 (0.62–1.86)	1.59 (0.79–3.20)	0.94 (0.49–1.81)	0.97 (0.51–1.85)
Eczema current	0.78 (0.47–1.30)	1.00 (0.69–1.45)	1.09 (0.56–2.15)	1.11 (0.60–2.05)	1.52 (0.74–3.12)	0.84 (0.40–1.74)	0.79 (0.39–1.63)
Diagnosed eczema	0.76 (0.52–1.13)	1.14 (0.81–1.59)	0.96 (0.53–1.74)	1.35 (0.78–2.34)	1.16 (0.60–2.23)	1.01 (0.53–1.92)	1.10 (0.58–2.08)

All models were adjusted for age, allergic history, home dampness, environmental tobacco smoke exposure and measurement season.

## Data Availability

Research data are not shared.

## Supplementary Materials

The following supporting information can be downloaded at: https://www.mdpi.com/article/10.3390/ijerph192114083/s1, Table S1: Mass–charge ratio and retention time of phthalate metabolites. Table S2: Mean and median concentrations (μg/L) of phthalate metabolites in different seasons. Table S3: Crude odds ratio (95% Confidence Intervals) of phthalate metabolites with children’s asthma and allergy in logistic regression models. Table S4: Stratification analysis on associations of phthalates metabolites with children’s asthma and allergy in winter–spring and summer–autumn seasons. Table S5: Median concentrations (μg/L) of phthalate metabolites in children’s urine in different studies. References [25,26,27,40,41,42,43,44] are cited in the supplementary materials.
