# Supplementary material for: Phthalate Metabolites in Urine of Chinese Children and Their Association with Asthma and Allergic Symptoms
Source: Int J Environ Res Public Health. 2022 Oct 28;19(21):14083. doi: 10.3390/ijerph192114083 (PMC9654528; doi:10.3390/ijerph192114083)
Supplement: Supplementary file 1 [file ijerph-19-14083-s001.zip › ijerph-1957315-supplementary.pdf]

# Supporting information

# Phthalate metabolites in urine of Chinese children and their association with asthma and allergic symptoms

**Yuxuan Zhao [1], Yuexia Sun [1,*], Changqi Zhu [1], Ying Zhang [2], Jing Hou [1], Qinghao Zhang [1] and Yeganeh Ataei [1]**

[1] Tianjin Key Laboratory of Indoor Air Environmental Quality Control, School of Environmental Science and Engineering, Tianjin University, Tianjin 300350, China

[2] School of Environmental Science and Engineering, Tianjin University, Tianjin 300350, China

* Correspondence: yuexiasun@tju.edu.cn

# Table of contents

**Table S1** Mass-charge ratio and retention time of phthalate metabolites

**Table S2** Mean and median concentrations (μg/L) of phthalate metabolites in different seasons

**Table S3** Crude odds ratio (95% Confidence Intervals) of phthalate metabolites with children's asthma and allergy in logistic regression models

**Table S4** Stratification analysis on associations of phthalates metabolites with children's asthma and allergy in winter-spring and summer-autumn seasons

**Table S5** Median concentrations (μg/L) of phthalate metabolites in children's urine in different studies

Table S1 Mass-charge ratio and retention time of phthalate metabolites

| | Mass-charge ratio | Retention time (min) | Internal standard compounds | Mass - charge ratio | Retention time (min) |
|---|---|---|---|---|---|
| MEP | 193/77 | 4.23 | $^{13}C_4$-MEP | 197/124 | 4.23 |
| MiBP | 221/77 | 5.75 | $^{13}C_4$-MnBP | 225/79 | 5.85 |
| MnBP | 221/71 | 5.85 | $^{13}C_4$-MnBP | 225/79 | 5.85 |
| MBzP | 255/77 | 6.24 | | | |
| MEHP | 277/134 | 8.09 | | | |
| MECPP | 307/113 | 5.68 | $^{13}C_4$-MECPP | 311/159 | 5.68 |
| MEHHP | 293/121 | 5.76 | | | |
| MEOHP | 297/77 | 5.97 | | | |

Table S2 Mean and median concentrations (μg/L) of phthalate metabolites in different seasons

| | | MEP | MiBP | MnBP | MEHP | MECPP | MEHHP | MEOHP |
|---|---|---|---|---|---|---|---|---|
| Spring | Mean | 61.38 | 24.88 | 23.02 | 7.16 | 44.73 | 11.12 | 8.67 |
| | Median | 13.93 | 31.16 | 17.99 | 7.48 | 54.47 | 13.87 | 8.44 |
| Summer | Mean | 52.29 | 31.66 | 41.56 | 8.22 | 33.24 | 14.16 | 9.80 |
| | Median | 45.32 | 32.53 | 36.85 | 8.06 | 33.86 | 16.66 | 12.33 |
| Autumn | Mean | 24.91 | 25.43 | 23.08 | 5.96 | 40.96 | 11.41 | 7.35 |
| | Median | 14.32 | 31.12 | 20.27 | 5.53 | 50.66 | 11.79 | 6.35 |
| Winter | Mean | 32.01 | 29.26 | 31.87 | 8.58 | 33.95 | 16.17 | 10.11 |
| | Median | 18.90 | 31.86 | 31.43 | 8.05 | 34.42 | 17.24 | 12.30 |
| P[a] | | 0.01 | 0.03 | 0.01 | 0.00 | 0.04 | 0.01 | 0.02 |

[a] Kruskal-Wallis test.

Table S3 Crude odds ratios (95% Confidence Intervals) of phthalate metabolites [a] with children's asthma and allergy in logistic regression models

| | MEP | MiBP | MnBP | MEHP | MECPP | MEHHP | MEOHP |
|---|---|---|---|---|---|---|---|
| Wheeze ever | 1.11 (0.95-1.31) | 1.06 (0.83-1.35) | 1.49 (0.91-2.44) | 1.06 (0.70-1.59) | 0.99 (0.56-1.74) | 1.16 (0.73-1.84) | 1.38 (0.82-2.34) |
| Wheeze current | 1.15 (0.97-1.36) | 1.25 (0.92-1.70) | 1.52 (0.81-2.83) | 1.19 (0.69-2.04) | 0.53 (0.24-1.17) | 1.31 (0.73-2.36) | 1.85 (0.90-3.78) |
| Current dry cough | 1.00 (0.85-1.19) | 1.04 (0.81-1.34) | 0.95 (0.56-1.62) | 0.93 (0.61-1.43) | 1.10 (0.62-1.96) | 1.27 (0.79-2.02) | 1.19 (0.70-2.02) |
| Diagnosed asthma | 0.93 (0.68-1.26) | 1.25 (0.95-1.63) | **1.98 (1.16-3.38)** | 1.35 (0.86-2.12) | 0.92 (0.49-1.73) | **2.04 (1.19-3.48)** | **2.16 (1.16-4.02)** |
| Rhinitis ever | 1.04 (0.89-1.22) | 0.93 (0.76-1.15) | 1.01 (0.66-1.55) | 1.00 (0.71-1.41) | 0.87 (0.54-1.38) | 0.83 (0.56-1.22) | 1.03 (0.67-1.58) |
| Rhinitis current | 0.92 (0.77-1.10) | 0.95 (0.78-1.17) | 0.96 (0.63-1.46) | 1.07 (0.77-1.50) | 1.06 (0.67-1.68) | 0.73 (0.49-1.08) | 0.82 (0.54-1.25) |
| Diagnosed rhinitis | 0.80 (0.54-1.19) | 1.12 (0.88-1.43) | 1.21 (0.74-1.96) | 1.09 (0.73-1.62) | 1.01 (0.59-1.75) | 1.19 (0.76-1.86) | 1.04 (0.63-1.71) |
| Eczema ever | 0.84 (0.65-1.07) | 0.88 (0.71-1.08) | 0.82 (0.54-1.25) | 0.85 (0.60-1.19) | 1.39 (0.87-2.22) | 0.75 (0.50-1.10) | 0.77 (0.50-1.18) |
| Eczema current | 0.88 (0.66-1.18) | 0.89 (0.71-1.13) | 0.96 (0.60-1.54) | 0.83 (0.56-1.21) | 1.26 (0.75-2.10) | 0.69 (0.44-1.09) | 0.65 (0.40-1.05) |
| Diagnosed eczema | 0.88 (0.72-1.09) | 1.03 (0.84-1.26) | 0.99 (0.65-1.51) | 0.99 (0.71-1.38) | 1.04 (0.66-1.64) | 0.90 (0.61-1.31) | 0.85 (0.55-1.29) |

[a] Concentrations of phthalate metabolites were coded as continues variables with an interquartile increase step. *p*-value less than 0.05 are indicated in bold for significance

Table S4 Stratification analysis on associations of phthalates metabolites with children's asthma and allergy in winter-spring and summer-autumn seasons

| | | MEP | MiBP | MnBP | MEHP | MECPP | MEHHP | MEOHP |
|---|---|---|---|---|---|---|---|---|
| Wheeze ever | Winter-spring | 1.21 (0.93-1.57) | 1.01 (0.84-1.21) | 1.25 (0.59-2.62) | 1.10 (0.53-2.29) | 1.18 (0.50-2.79) | 1.13 (0.74-1.71) | 1.65 (0.81-3.33) |
| | Summer-autumn | 0.97 (0.54-1.75) | 0.89 (0.52-1.55) | 1.52 (0.66-3.45) | 1.05 (0.48-2.31) | 0.88 (0.35-2.24) | 0.77 (0.27-2.21) | 0.89 (0.30-2.65) |
| Wheeze current | Winter-spring | 1.26 (0.91-1.74) | 1.07 (0.86-1.35) | 1.94 (0.74-5.03) | 1.24 (0.48-3.16) | 0.58 (0.18-1.88) | 1.14 (0.69-1.88) | 2.67 (0.97-7.37) |
| | Summer-autumn | 0.53 (0.13-2.21) | 1.28 (0.63-2.57) | 1.13 (0.34-3.78) | 1.83 (0.59-5.70) | 0.53 (0.13-2.11) | 0.98 (0.24-3.97) | 0.89 (0.20-4.04) |
| Current dry cough | Winter-spring | 0.95 (0.73-1.22) | 1.03 (0.86-1.24) | 1.24 (0.56-2.74) | 1.10 (0.53-2.26) | 0.88 (0.36-2.17) | 1.35 (0.85-2.12) | 1.52 (0.74-3.14) |
| | Summer-autumn | 1.50 (0.87-2.59) | 1.15 (0.67-1.97) | 1.12 (0.44-2.82) | 0.59 (0.23-1.51) | 0.92 (0.33-2.61) | 0.93 (0.29-3.01) | 0.65 (0.18-2.39) |
| Diagnosed asthma | Winter-spring | 0.84 (0.45-1.55) | 1.22 (0.95-1.57) | 2.06 (0.89-4.78) | 1.22 (0.54-2.75) | 1.29 (0.49-3.41) | 1.73 (0.97-3.11) | 2.18 (0.95-4.96) |
| | Summer-autumn | 1.50 (0.85-2.65) | 0.81 (0.41-1.59) | 1.95 (0.79-4.85) | 2.08 (0.76-5.66) | 0.48 (0.13-1.77) | 1.59 (0.46-5.49) | 1.79 (0.48-6.68) |
| Rhinitis ever | Winter-spring | 1.07 (0.88-1.29) | 0.89 (0.75-1.06) | 0.90 (0.47-1.74) | 0.75 (0.40-1.42) | 1.08 (0.50-2.34) | 0.76 (0.52-1.13) | 0.98 (0.54-1.76) |
| | Summer-autumn | 1.16 (0.79-1.71) | 1.10 (0.76-1.61) | 1.27 (0.65-2.47) | 1.09 (0.58-2.06) | 0.61 (0.29-1.29) | 0.81 (0.36-1.83) | 0.95 (0.41-2.21) |
| Rhinitis current | Winter-spring | 0.88 (0.65-1.19) | 0.91 (0.78-1.07) | 0.86 (0.46-1.62) | 0.86 (0.47-1.59) | 1.36 (0.65-2.84) | 0.66 (0.43-1.00) | 0.69 (0.39-1.22) |
| | Summer-autumn | 1.15 (0.79-1.70) | 1.06 (0.73-1.55) | 1.10 (0.57-2.13) | 1.22 (0.65-2.30) | 0.79 (0.38-1.61) | 0.74 (0.33-1.65) | 0.78 (0.34-1.80) |
| Diagnosed rhinitis | Winter-spring | 0.55 (0.24-1.28) | 1.06 (0.88-1.28) | 1.28 (0.59-2.78) | 0.91 (0.42-1.96) | 1.30 (0.55-3.04) | 1.02 (0.67-1.55) | 0.78 (0.40-1.51) |
| | Summer-autumn | 1.11 (0.60-2.04) | 0.91 (0.50-1.65) | 1.12 (0.45-2.77) | 1.36 (0.58-3.21) | 0.70 (0.24-2.01) | 1.27 (0.43-3.80) | 1.25 (0.39-3.99) |
| Eczema ever | Winter-spring | 0.87 (0.65-1.16) | 1.07 (0.91-1.26) | 1.27 (0.68-2.37) | 0.96 (0.53-1.75) | 0.81 (0.39-1.66) | 1.04 (0.72-1.50) | 0.95 (0.55-1.64) |
| | Summer-autumn | 0.83 (0.56-1.23) | 0.61 (0.37-1.00) | 0.49 (0.23-1.04) | 0.77 (0.41-1.47) | 2.25 (0.97-5.24) | 0.52 (0.23-1.22) | 0.61 (0.26-1.45) |
| Eczema current | Winter-spring | 0.90 (0.63-1.27) | 0.90 (0.75-1.08) | 0.97 (0.48-1.97) | 0.79 (0.40-1.55) | 0.83 (0.38-1.85) | 0.76 (0.49-1.18) | 0.72 (0.39-1.32) |
| | Summer-autumn | 0.92 (0.57-1.49) | 0.96 (0.60-1.53) | 0.89 (0.40-1.97) | 0.67 (0.32-1.40) | **2.49 (1.04-5.98)** | 0.68 (0.27-1.72) | 0.42 (0.15-1.19) |
| Diagnosed eczema | Winter-spring | 0.91 (0.72-1.14) | 1.01 (0.86-1.18) | 1.23 (0.66-2.30) | 1.00 (0.55-1.81) | 0.98 (0.48-2.00) | 1.03 (0.71-1.48) | 0.93 (0.54-1.60) |
| | Summer-autumn | 0.97 (0.66-1.43) | 1.15 (0.79-1.67) | 0.79 (0.41-1.53) | 1.24 (0.67-2.30) | 0.89 (0.44-1.78) | 0.72 (0.33-1.60) | 0.77 (0.34-1.75) |

Odds ratios were adjusted for age, gender, allergic history, home dampness and environmental tobacco smoke exposure. Concentrations of phthalate metabolites were coded as continuous variables with an interquartile increase step. *p*-value less than 0.05 are indicated in bold for significance

Table S5 Median concentrations (μg/L) of phthalate metabolites in children's urine in different studies

| References | Country | Sampling time | Sample size | MEP | MiBP | MnBP | MBzP | MEHP | MECPP | MEHHP | MEOHP |
|---|---|---|---|---|---|---|---|---|---|---|---|
| (Lewis, 2013) | Mexico | 2010 | 53 (boys) | 62.7 | 11 | 91.2 | 5.6 | 7.5 | 71.8 | 45.4 | 20.9 |
| | | | 55 (girls) | 78.4 | 12.7 | 98 | 5 | 6.4 | 69.8 | 42.5 | 18.7 |
| (Koch, 2011) | Germany | 2007 | 111 | / | 42.8 | 36.8 | 7.2 | 4.7 | 28.4 | 17.4 | 15.1 |
| (Fromme, 2013) | Germany | 2011-2012 | 663 | 14.5 | 44.7 | 32.4 | 11.6 | / | / | 16.5 | 17.9 |
| (Bertelsen, 2013) | Norway | 2001-2004 | 623 | 56.7 | 49.2 | 138 | 29.3 | 7.8 | 98.2 | 76.6 | 49.7 |
| (Boas, 2010) | Denmark | 2006-2007 | 503 (boys) | 21 | 130 | | 17 | 4.5 | 30 | 37 | 19 |
| | | | 342 (girls) | 21 | 121 | | 12 | 3.6 | 27 | 31 | 16 |
| (Langer, 2014) | Denmark | 2008-2009 | 441 | 16.6 | 72.2 | 80.1 | 13 | 4.7 | 34.5 | 33.2 | 17.6 |
| (Liao, 2018) | China | 2013-2014 | 434 | 10.1 | 46.2 | 58.1 | 0.2 | 4.5 | 39 | 24.7 | 14.5 |
| (Gong, 2015) | China | 2013-2014 | 39 (summer) | 23.8 | 78.2 | 224 | / | 6.5 | 68.4 | 25.2 | 21.6 |
| | | | 39 (winter) | 15.3 | 54.1 | 120 | / | 9.3 | 70.9 | 47.9 | 28.2 |
| (Wang, 2013) | China | 2012 | 259 | 15.9 | 37.4 | 47.2 | 0.05 | 21.1 | 29.8 | 15.7 | 22.9 |
| This study | China | 2013-2016 | 243 | 18.4 | 31.6 | 26.2 | 0.09 | 7.5 | 46.1 | 16.7 | 9.6 |

## References

Boas, M., Frederiksen, H., Feldt-Rasmussen, U., Skakkebaek, N.E., Hegedus, L., Hilsted, L., Juul, A., Main, K.M., 2010. Childhood exposure to phthalates: Associations with thyroid function, insulin-like growth factor i, and growth. *Environmental Health Perspectives*. 10, 1458-64.

Fromme, H., Lahrz, T., Kraft, M., Fembacher, L., Dietrich, S., Sievering, S., Burghardt, R., Schuster, R., Bolte, G., Volkel, W., 2013. Phthalates in German daycare centers: Occurrence in air and dust and the excretion of their metabolites by children (lupe 3). *Environment International*. 64-72.

Gong, M., Weschler, C.J., Liu, L., Shen, H., Huang, L., Sundell, J., Zhang, Y., 2015. Phthalate metabolites in urine samples from beijing children and correlations with phthalate levels in their handwipes. *Indoor Air*. 6, 572-81.

Koch, H.M., Wittassek, M., Bruning, T., Angerer, J., Heudorf, U., 2011. Exposure to phthalates in 5-6 years old primary school starters in Germany-a human biomonitoring study and a cumulative risk assessment. *International Journal of Hygiene and Environmental Health*. 3, 188-95.

Langer, S., Beko, G., Weschler, C.J., Brive, L.M., Toftum, J., Callesen, M., Clausen, G., 2014. Phthalate metabolites in urine samples from Danish children and correlations with phthalates in dust samples from their homes and daycare centers. *International Journal of Hygiene and Environmental Health*. 1, 78-87.

Lewis, R.C., Meeker, J.D., Peterson, K.E., Lee, J.M., Pace, G.G., Cantoral, A., Tellez-Rojo, M.M., 2013. Predictors of urinary bisphenol a and phthalate metabolite concentrations in Mexican children. *Chemosphere*. 10, 2390-8.

Liao, C.X., Liu, W., Zhang, J.L., Shi, W.M., Wang, X.Y., Cai, J., Zou, Z.J., Lu, R.C., Sun, C.J., Wang, H., Huang, C., Zhao, Z.H., 2018. Associations of urinary phthalate metabolites with residential characteristics, lifestyles, and dietary habits among young children in Shanghai, China. *Science of the Total Environment*. 1288-97.

Wang, H.X., Zhou, Y., Tang, C.X., He, Y.H., Wu, J.G., Chen, Y., Jiang, Q.W., 2013. Urinary phthalate metabolites are associated with body mass index and waist circumference in Chinese school children. *Plos One*. 2.
